# Association of Receiving a Fourth Dose of the BNT162b Vaccine With SARS-CoV-2 Infection Among Health Care Workers in Israel

**DOI:** 10.1001/jamanetworkopen.2022.24657

**Published:** 2022-08-02

**Authors:** Matan J. Cohen, Yonatan Oster, Allon E. Moses, Avishay Spitzer, Shmuel Benenson, and the Israeli-Hospitals 4th Vaccine Working Group

**Affiliations:** 1 Clalit Health Services, Jerusalem District, Israel; 2 Department of Clinical Microbiology and Infectious Diseases, Hadassah-Hebrew University Medical Center, Jerusalem, Israel; 3 Department of Oncology, Tel Aviv Sourasky Medical Center, Tel Aviv, Israel; 4 Unit for Infection Prevention and Control, Shaare-Zedek Medical Center, Jerusalem, Israel

## Abstract

**Importance:**

Despite the high 3-dose vaccination rate among health care workers (HCWs) in Israel, a high rate of SARS-CoV-2 breakthrough infections in this group was observed during the Omicron wave. As a result, the Israeli Ministry of Health decided to recommend a fourth vaccine dose to medical staff.

**Objective:**

To evaluate the benefit of a fourth BNT162b2 vaccine dose on the breakthrough infection rate among HCWs.

**Design, Setting, and Participants:**

This multicenter cohort study was performed in January 2022, the first month of the 4-dose vaccination campaign, during a surge of the Omicron variant wave. All health care workers at 11 general hospitals in Israel who had been vaccinated with 3 doses up to September 30, 2021, and had not contracted COVID-19 before the vaccination campaign were included.

**Exposures:**

Vaccination with a fourth dose of the BNT162b2 vaccine during January 2022.

**Main Outcomes and Measures:**

Breakthrough COVID-19 infections in 4-dose recipients vs 3-dose recipients measured by a polymerase chain reaction test result positive for SARS-CoV-2. Health care workers were tested based on symptoms or exposure.

**Results:**

A total of 29 611 Israeli HCWs (19 381 [65%] female; mean [SD] age, 44 [12] years) had received 3 vaccine doses between August and September 2021; of these, 5331 (18%) received the fourth dose in January 2022 and were not infected by the first week after vaccination. Overall breakthrough infection rates were 368 of 5331 (7%) in the 4-dose group and 4802 of 24280 (20%) in the 3-dose group (relative risk, 0.35; 95% CI, 0.32-0.39). Similar reductions were found in a matched analysis by the exact day of receiving the third vaccine (relative risk, 0.61; 95% CI, 0.54-0.71) and in a time-dependent Cox proportional hazards regression model (adjusted hazard ratio, 0.56; 95% CI, 0.50-0.63). In both groups, no severe disease or death occurred.

**Conclusions and Relevance:**

In this cohort study, the fourth BNT162b2 vaccine dose resulted in a reduced breakthrough infection rate among hospital staff. This reduction was lower than that observed after the third dose; nevertheless, considering the high infectivity of the Omicron variant, which led to critical medical staff shortages, a fourth vaccine dose should be considered to mitigate the infection rate among HCWs.

## Key Points

**Question:**

Was there a benefit of vaccinating health care workers with a fourth dose of BNT162b2 vaccine during the Omicron variant outbreak of the COVID-19 pandemic?

**Findings:**

In this multicenter cohort study of 29 611 health care workers in Israel, the breakthrough infection rate among those who received 4 doses was 6.9% compared with 19.8% in those who received 3 doses.

**Meaning:**

These findings suggest that a fourth vaccine dose was effective in preventing breakthrough COVID-19 infections in health care workers, helping to maintain the function of the health care system during the pandemic.

## Teaser

This cohort study of health care workers in Israel evaluates whether a fourth BNT162b2 vaccine dose lowered breakthrough infection rates of SARS-CoV-2.

## Introduction

In December 2021, the fifth COVID-19 wave started in Israel, caused mostly by the Omicron variant and affecting the unvaccinated and vaccinated populations.^1,2^ Israel was the first country to administer a third vaccine dose to the entire adult population, beginning in August 2021. Ninety percent of adults in Israel, including more than 95% of health care workers (HCWs),^3^ received 3 doses of the BNT162b2 vaccine until September 2021. This third dose (booster) vaccine markedly reduced the rate of breakthrough infections, severe disease, and mortality during the Delta variant wave.^4^ Despite this high vaccination rate, the highly infectious Omicron variant caused a significant number of breakthrough infections among the thrice-vaccinated population. Following the success and safety of the third dose in preventing infection and severe disease, and assuming waning immunity of the third dose, the Israeli Ministry of Health recommended a voluntary fourth BNT162b2 dose to adults older than 60 years, those who were immunocompromised, and HCWs. During the peak of Omicron activity, we compared breakthrough infection rates between HCWs who had received 3 vs 4 doses of the BNT162b2 vaccine.

## Methods

### Design and Settings

Eleven Israeli hospitals participated in the initial study: all are academic centers, 5 of which are tertiary care centers. Health care workers were vaccinated with Pfizer's BNT162b2 messenger RNA vaccine. In total, the participating centers included approximately half of the total acute care beds in Israel and cover all areas of Israel. The study was approved by the local ethics committee of each center, including waiver of informed consent because all the data were deidentified before being added to the study database. This study followed the Strengthening the Reporting of Observational Studies in Epidemiology (STROBE) reporting guideline.

### Data Collection

The study cohort included all HCWs who have been vaccinated with 3 doses of the BNT162b2 vaccine and had not contracted COVID-19 any time before the vaccination campaign (January 2, 2022). We collected data on anonymized personnel demographic characteristics (sex, age group, and profession) and vaccination and breakthrough infection dates for all participants until January 31, 2022. Because 99% of 4-dose recipients received their third dose during August and September 2021, we limited the analysis and comparison of 3-dose and 4-dose recipients to the HCWs who were vaccinated with the third dose only during these months. Comparison of the sex, age, and profession of the HCWs who were vaccinated during August and September 2021 to the entire cohort yielded similar distributions.

### Data Analysis

Workers were tested by polymerase chain reaction on nasopharyngeal swabs either because of symptoms or after exposure; no systematic testing was performed. We calculated breakthrough infection rates occurring during January 2022 in 4-dose recipients (at least 1 week after vaccination) vs 3-dose recipients. We then generated rate ratios for the entire cohort and per subgroups: hospital, sex, age group (<40 years, 40-59 years, and ≥60 years) and profession (physician, nursing, or other).

To establish the robustness of the results, we performed 2 additional analyses: matched analysis and regression modeling. In the matched analysis (1:1 matching), for each 4-dose vaccinated participant, a 3-dose vaccinated participant was matched by sex, age group, profession, hospital, and date of receiving the third vaccine dose (exact date matching). The matched 3-dose participant was selected only if not infected by the date the matched 4-dose partner was vaccinated. If multiple potential matching partners were found, one was randomly selected. We used the McNemar test for the matched analysis, and 95% CIs for the relative risk were calculated using the Wald method. We produced an inverse Kaplan-Meier survival curve for the crude and matched analyses, demonstrating the proportion of HCWs who contracted COVID-19 in each group (3- and 4-dose groups) during January 2022.

In the regression modeling, the timing of receiving the fourth dose was at the participants’ discretion and could have taken place at any day after the fourth vaccine was recommended for HCWs. Therefore, exposure status to the fourth dose varied over time. To account for the changing exposure status throughout the study period, a Cox proportional hazards regression analysis with exposure status as a time-varying covariate was conducted, evaluating the hazard ratio associated with fourth dose exposure status among HCWs who received 3 vaccine doses during August and September 2021. All participants were defined as unexposed at study initiation and as exposed 7 days after receiving the fourth dose. Participants were censored because of a confirmed SARS-CoV-2 infection or at the end of the study period. The Cox proportional hazards regression model was adjusted for sex, age group, profession, hospital, and month of receiving the third vaccine dose. All covariates were tested with Schoenfeld residuals to evaluate the assumption of hazard proportionality. Nonproportional hazards were handled using stratification. This unmatched model was followed with a model that included complete matching as described in the previous analysis.

### Statistical Analysis

In the matched analysis, we compared the crude curves using the log-rank test and the matched data with the Prentice-Wilcoxon test and the Gehan test for comparison of paired data. These analyses were performed with WINPEPI software, version 11.65 (J. Abramson). The statistical analyses for the regression modeling were performed using R software, version 4.0.3 (R Foundation for Statistical Computing). The Cox proportional hazards regression model was computed using the survival package. Other packages used were dplyr, tibble, ggplot2, and survminer. A 2-sided *P* < .05 was considered to be statistically significant.

## Results

Overall, in the 11 hospitals participating in this study between August and September 2021, a total of 29 611 HCWs (19 381 [65%] female and 10 230 [35%] male; mean [SD] age, 44 [12] years) had received their third vaccine dose. Of these HCWs, 7370 (25%) were physicians, 8946 (30%) were nurses, and 13 295 (45%) were of other professions (Table 1). A higher proportion of male HCWs compared with female HCWs chose to receive the fourth dose (2503 of 10 230 [25%] vs 3016 of 19 381 [16%], *P* < .001). Older HCWs chose to receive the fourth dose more frequently than younger ones (≥60 years, 1749 of 4172 [42%]; 40-59 years, 2605 of 13 898 [19%]; and <40 years, 1165 of 11 541 [10%]; *P* < .001). Physicians were vaccinated more frequently (1887 of 7370 [26%] than the nursing staff (1215 of 8946 [14%] and other professions (2417 of 13 295 [18%]) (*P* < .001). Characteristics of the HCWs were similar in all participating hospitals (Table 2).

**Table 1.** Characteristics of 29 611 HCWs From 11 Hospitals in Israel by Number of Vaccine Doses^a^

Characteristic	No./total No. (%)			*P* value
	Total (N = 29611)	3-Dose group (n = 24 092)	4-Dose group (n = 5519)	
Sex				
Female	19 381 (65)	16 365/19 381 (84)	3016/19 381 (16)	<.001
Male	10 230 (35)	7727/10 230 (75)	2503/10 230 (25)	
Age group, y				
<40	11 541 (39)	10 376/11 541 (90)	1165/11 541 (10)	<.001
40-59	13 898 (47)	11 293/13 898 (81)	2605/13 898 (19)	
≥60	4172 (14)	2423/4172 (58)	1749/4172 (42)	
Profession				
Physician	7370 (25)	5483/7370 (74)	1887/7370 (26)	<.001
Nursing	8946 (30)	7731/8946 (86)	1215/8946 (14)	
Other	13 295 (45)	10 878/13 295 (82)	2417/13 295 (18)	

Abbreviation: HCW, health care worker.

^a^ A total of 188 HCWs who were vaccinated with the fourth dose contracted COVID-19 within 1 week of vaccination and therefore were included in the 3-dose group in the analysis.

**Table 2.** Number of Participating HCWs by Hospital and Profession Who Received 3 or 4 Doses of the BNT162b2 Vaccine^a^

Variable	No. (%)											
	BMC (n = 1382)	BPMCP (n = 1215)	BZMC (n = 1607)	HHUMC (n = 3900)	KMC (n = 1872)	MMC (n = 2581)	RHCC (n = 4457)	SMC (n = 2451)	SZMC (n = 2864)	TASMC (n = 5595)	WMC (n = 1723)	Total (N = 29 611)
Profession												
Physician	351 (25)	321 (26)	382 (24)	807 (21)	470 (25)	623 (24)	1022 (23)	618 (26)	827 (29)	1467 (26)	482 (28)	7370 (25)
Nursing	522 (38)	369 (30)	477 (30)	1167 (30)	690 (37)	955 (37)	1051 (24)	857 (35)	1005 (35)	1241 (22)	612 (36)	8946 (30)
Other	509 (37)	525 (43)	748 (47)	1926 (49)	712 (38)	1003 (39)	2384 (53)	940 (39)	1032 (36)	2887 (52)	629 (37)	13 295 (45)
No. of vaccine doses												
3	1228 (89)	1100 (91)	1282 (80)	3038 (78)	1508 (81)	2019 (78)	3787 (85)	2070 (86)	2166 (76)	4362 (78)	1532 (89)	24 092 (81)
4	154 (11)	115 (9)	325 (20)	862 (22)	364 (19)	562 (22)	670 (15)	345 (14)	698 (24)	1233 (22)	191 (11)	5519 (19)

Abbreviations: BMC, Barzilai Medical Center; BPMCP, Baruch Padeh Medical Center Poriya; BZMC, Bnai Zion Medical Center; HCW, health care worker; HHUMC, Hadassah Hebrew University Medical Center; KMC, Kaplan Medical Center; MMC, Meir Medical Center; RHCC, Rambam Health Care Campus; SMC, Shamir Medical Center; SZMC, Shaare Zedek Medical Center; TASMC, Tel Aviv Sourasky Medical Center; WMC, Wolfson Medical Center.

^a^ A total of 188 HCWs who were vaccinated with the fourth dose contracted COVID-19 within 1 week of vaccination and therefore were included in the 3-dose group in the analysis. We included only HCWs who received the third vaccine dose between August 1 and September 30, 2021.

**Figure 1.**
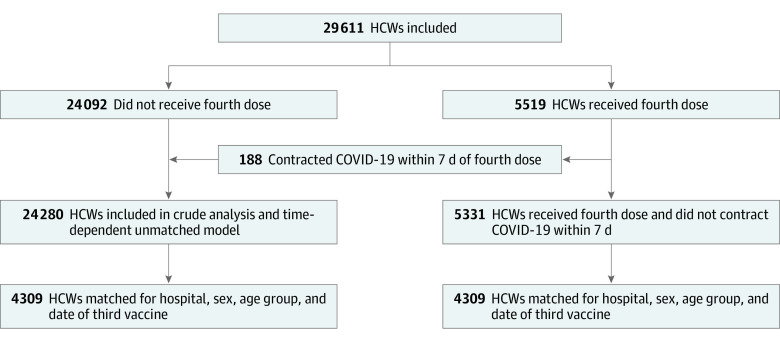
Flowchart of the Analyses of Breakthrough Infections Among Recipients of the 3- and 4-Dose Vaccines in Health Care Workers (HCWs) in 11 Hospitals in Israel

During January 2022, a total of 5519 HCWs received the fourth dose. Of these HCWs, 188 contracted COVID-19 within 1 week of vaccination and therefore were included in the 3-dose group, creating a 4-dose group of 5331 HCWs (Figure 1).

Breakthrough infection rates were 368 of 5331 (7%) in the 4-dose group and 4802 of 24 280 (20%) in the 3-dose group. The relative risk was 0.35 (95% CI, 0.32-0.39) for crude analysis and 0.61 (95% CI, 0.54-0.71) in the matched analysis. The adjusted hazard ratio in the Cox proportional hazards regression model was 0.56 (95% CI, 0.50-0.63).

**Figure 2.**
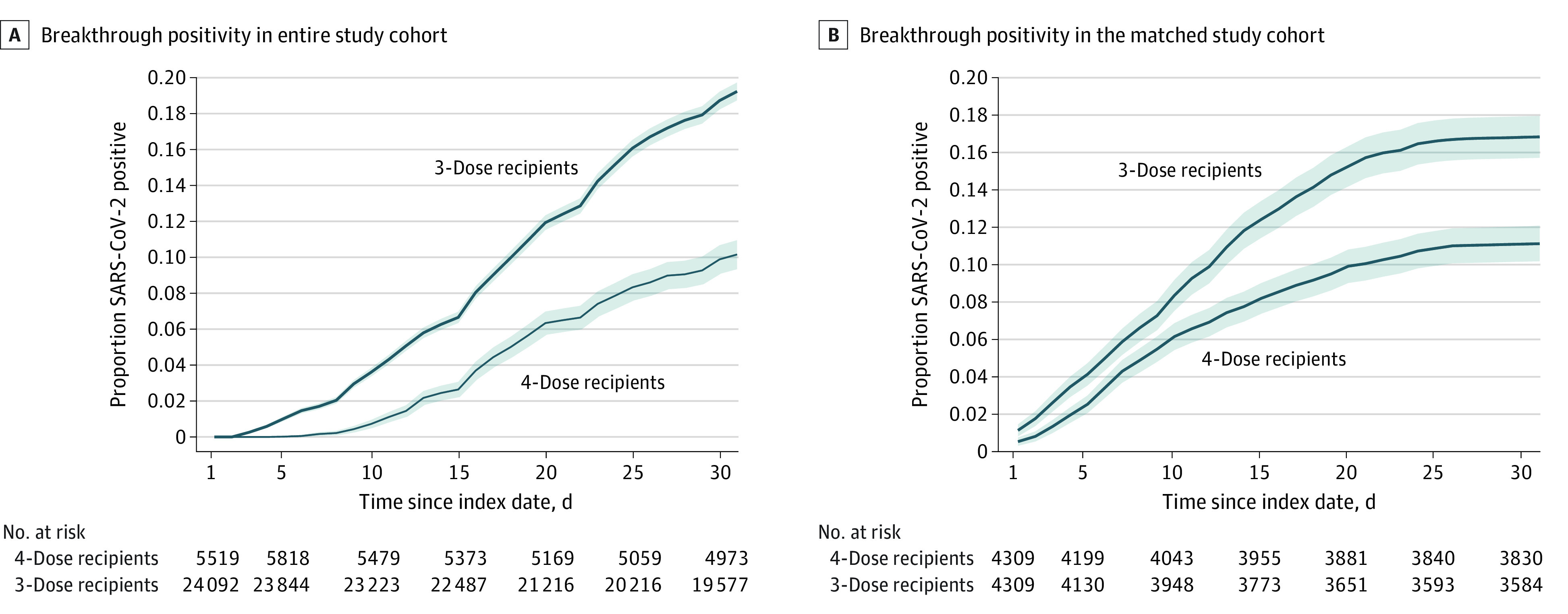
Morbidity Curves of the Cumulative SARS-CoV-2 Positivity Rate Estimate Among Health Care Workers Who Received 4 Vaccine Doses vs 3 Vaccine Doses For each matched pair, the index date was the day the worker received the fourth vaccine dose (*P* < .001). Gray shading indicates 95% CIs.

Overall, the effect of the fourth dose on the COVID-19 infection rate was consistent over crude, matched, and modeled analyses in all subgroups (Table 3). Kaplan-Meier curves of the crude and matched data are presented in Figure 2. In both groups, no severe disease or death occurred.

**Table 3.** Breakthrough Infection Rates Among 4-Dose and 3-Dose Vaccinated Health Care Workers

Characteristic	No. infected/No. at risk (%)		Crude analysis, RR (95% CI)	Time-dependent model, adjusted HR (95% CI)	Matched comparisons, RR (95% CI)
	3-Dose group (n = 24 280)	4-Dose group (n = 5331)			
All	4802/24 280 (20)	368/5331 (7)	0.35 (0.32-0.39)	0.56 (0.50-0.63)	0.61 (0.54-0.71)
Sex					
Male	1415/7804 (18.)	154/2426 (6)	0.35 (0.30-0.41)	0.56 (0.49-0.65)	0.66 (0.56-0.79)
Female	3387/16 476 (21)	214/2905 (7)	0.36 (0.31-0.41)	0.55 (0.46-0.66)	0.57 (0.46-0.70)
Age group, y					
<40	2044/10 429 (20)	81/1112 (7)	0.37 (0.30-0.46)	0.57 (0.45-0.72)	0.62 (0.48-0.81)
40-59	466/2466 (19)	106/1706 (6)	0.33 (0.27-0.40)	0.56 (0.48-0.65)	0.58 (0.48-0.71)
≥60	2292/11 385 (20)	181/2513 (7)	0.36 (0.31-0.41)	0.55 (0.45-0.68)	0.73 (0.54-0.99)
Profession					
Physician	928/5538 (17)	114/1832 (6)	0.37 (0.31-0.45)	0.60 (0.49-0.74)	0.63 (0.49-0.82)
Nursing	1927/7790 (25)	114/1156 (10)	0.40 (0.33-0.48)	0.58 (0.48-0.7)	0.65 (0.51-0.83)
Other	1947/10 952 (18)	140/2343 (6)	0.34 (0.28-0.40)	0.53 (0.45-0.63)	0.59 (0.48-0.74)
Hospital					
BMC	53/1228 (4)	8/154 (5)	1.20 (0.58-2.48)	1.65 (0.74-3.7)	1.75 (0.51-5.98)
BPMCP	225/1106 (20)	10/109 (9)	0.45 (0.25-0.82)	0.71 (0.37-1.36)	1.00 (0.35-2.85)
BZMC	234/1289 (18)	16/318 (5)	0.28 (0.17-0.45)	0.41 (0.24-0.69)	0.72 (0.36-1.44)
HHUMC	891/3076 (29)	87/824 (11)	0.36 (0.30-0.45)	0.57 (0.46-0.71)	0.68 (0.51-0.91)
KMC	320/1516 (21)	20/356 (6)	0.27 (0.17-0.41)	0.48 (0.3-0.77)	0.61 (0.31-1.18)
MMC	364/2040 (18)	22/541 (4)	0.23 (0.15-0.35)	0.51 (0.34-0.77)	0.37 (0.20-0.67)
RHCC	669/3800 (18)	30/657 (5)	0.26 (0.18-0.37)	0.39 (0.27-0.56)	0.40 (0.26-0.61)
SMC	471/2090 (22)	21/325 (6)	0.29 (0.19-0.44)	0.50 (0.32-0.78)	0.52 (0.30-0.88)
SZMC	493/2195 (22)	50/669 (8)	0.33 (0.25-0.44)	0.54 (0.4-0.73)	0.68 (0.48-0.96)
TASMC	781/4393 (18)	98/1202 (8)	0.46 (0.38-0.56)	0.74 (0.6-0.92)	0.73 (0.56-0.96)
WMC	301/1547 (20)	6/176 (3)	0.18 (0.08-0.39)	0.56 (0.31-1.01)	0.53 (0.23-1.26)

Abbreviations: BMC, Barzilai Medical Center; BPMCP, Baruch Padeh Medical Center Poriya; BZMC, Bnai Zion Medical Center; HHUMC, Hadassah Hebrew University Medical Center; HR, hazard ratio; KMC, Kaplan Medical Center; MMC, Meir Medical Center; RHCC, Rambam Health Care Campus; RR, relative risk; SMC, Shamir Medical Center; SZMC, Shaare Zedek Medical Center; TASMC, Tel Aviv Sourasky Medical Center; WMC, Wolfson Medical Center.

## Discussion

In this multicenter cohort study of HCWs in 11 general hospitals in Israel, we found that 4-dose vaccine recipients had a lower risk of acquiring COVID-19 than 3-dose recipients during the peak Omicron variant wave. The benefit of the fourth vaccine was significant in subgroup analyses (sex, age group, and profession) and in most participating hospitals (Table 3). These results were consistent and significant in the matched analysis and the Cox proportional hazards regression model as well, albeit less prominent. This finding might be explained by the smaller group size in these analyses. In other studies^5,6^ that examined the effect of the fourth dose in Israel, in the elderly population, a similar benefit was reported.

The third vaccine dose had markedly reduced the COVID-19 infection rate during the Delta variant outbreak.^7^ The fourth dose reduced the infection rate as well, but to a lesser extent. In a study from Israel,^8^ the antibody increase observed after the fourth dose was less prominent than after the third dose. This increase was not found in another multicenter study^9^ or in immunocompromised individuals.^10,11^

As opposed to the close-to-universal adherence to the third vaccine among HCWs in Israel,^12^ the overall fourth-dose vaccination rate in our cohort was only 19%. This finding reflects the doubt among HCWs regarding the necessity of a second booster. The common assumption was that the combination of reduced virulence of the Omicron variant and the protection given by the first 3 vaccine doses created no added value for the fourth vaccine. However, when considering vaccination of HCWs, the infection rate is equally important to complications or mortality rates because quarantine and isolation of a large number of HCWs may impair the ability of the health system to function, as observed in the beginning of the pandemic.^13^

Compared with HCWs, the vaccination rate among persons older than 60 years and immunocompromised persons (who were recommended to be vaccinated by the fourth dose in Israel) was much higher.^1^ This finding is probably related to the higher perceived risk in these populations. This belief was also reflected in the higher vaccination rate among older HCWs in our cohort (Table 1). In addition, vaccination rates were higher among physicians than among nurses and other professions. Other studies^14,15^ have also found lower vaccination rates among nurses for COVID-19 as well as for influenza virus.

### Limitations

Our study has some limitations. First, HCWs were not tested on a routine basis; therefore, some infections might have been missed. Second, because the decision to receive the fourth vaccine was on a voluntary basis, it is possible that those who chose to receive the fourth dose were more careful to avoid getting infected. The short follow-up period is another limitation of this study; however, the overall infection rate had decreased rapidly in the following weeks, and thus we assume that our results represent a true protective effect.

## Conclusions

This cohort study found that the fourth BNT162b2 vaccine dose was associated with a significant decrease in the COVID-19 infection rate among HCWs in Israel. In future COVID-19 waves, an additional vaccine dose should be considered as an effective method to preserve the function of the health system.

## Notes

Supplement.
Nonauthor Collaborators
