# Supplementary material for: Association of Receiving a Fourth Dose of the BNT162b Vaccine With SARS-CoV-2 Infection Among Health Care Workers in Israel
Source: JAMA Netw Open. 2022 Aug 2;5(8):e2224657. doi: 10.1001/jamanetworkopen.2022.24657 (PMC9346545; doi:10.1001/jamanetworkopen.2022.24657)
Supplement: Supplement. — Nonauthor Collaborators [file jamanetwopen-e2224657-s001.pdf]

Supplemental Online Content: Nonauthor Collaborators
*First name, last name, and suffix (if applicable) are required and will appear in PubMed.

***Group Name(s): The Israeli-Hospitals 4th Vaccine Working Group**

***First Name and Middle Initial(s)**	***Last Name**	***Suffix (eg, Jr, III)**	Academic Degrees	Institution	Location (city, state/province, country)	Role or Contribution, eg, chair, principal investigator	Group (if more than 1 Group listed in the byline) and/or Subgroup (eg, Steering Committee)
Asala	Abu-Ahmad			Infectious diseases unit, Bnai-Zion Medical Center			
Yoel	Angel			Tel Aviv Sourasky Medical Center; Faculty of Medicine, Tel Aviv University, Israel			
Ronen	Ben-Ami			Tel Aviv Sourasky Medical Center; Faculty of Medicine, Tel Aviv University, Israel			
Debby	Ben-David			Infection Control Unit, Edith Wolfson Medical Center; Faculty of Medicine, Tel Aviv University, Tel Aviv, Israel			
Inon	Buda			General Management, Hadassah-Hebrew University Medical Center			
Michal	Chowers			Meir Medical Center; Faculty of Medicine, Tel-Aviv University, Tel-Aviv, Israel			
Ayelet	Elbirt			Kaplan Medical Center, Clalit Health Services, Israel, Faculty of Medicine, Hebrew University Jerusalem, Israel			
Khetam	Hussein			Rambam Health Care Campus, Haifa, Israel; The Ruth & Bruce Rappaport Faculty of Medicine, Technion–Israel Institute of Technology			
Chezi	Levi			Barzilai Medical Center; Ben Gurion University, Beer-Sheva, Israel			
Yasmin	Maor			Infectious Disease Unit, Edith Wolfson Medical Center; Faculty of Medicine, Tel Aviv University, Tel Aviv, Israel			
Ran	Nir-Paz			Department of Clinical Microbiology and Infectious Diseases, Hadassah-Hebrew University Medical Center; Faculty of Medicine, Hebrew University Jerusalem, Israel			
Alona	Paz			Infectious Diseases Unit, Bnai-Zion Medical Center			
Esther	Saiag			Tel Aviv Sourasky Medical Center; Faculty of Medicine, Tel Aviv University, Israel			
Shlomo	Maayan			Barzilai Medical Center; Ben Gurion University, Beer-Sheva, Israel			
Yael	Shachor-Meyouhas			Rambam Health Care Campus, Haifa, Israel; The Ruth & Bruce Rappaport Faculty of Medicine, Technion–Israel Institute of Technology			
Pnina	Shitrit			Meir Medical Center; Faculty of Medicine, Tel-Aviv University, Tel-Aviv, Israel			
Miriam	Weinberger			Shamir (Assaf Harofeh) Medical Center; Faculty of Medicine, Tel Aviv University, Tel Aviv, Israel			
Yonit	Wiener-Well			Infectious Diseases Unit, Sharee-Zedek Medical Center; Faculty of Medicine, Hebrew University Jerusalem, Israel			
Adi	Zaidman Shimshovitz			Infectious Diseases Unit, Baruch Padeh Medical Center; the Azrieli Faculty of Medicine, Bar-Ilan University, Safed, Israel			
Eli	Sprecher			Tel Aviv Sourasky Medical Center; Faculty of Medicine, Tel Aviv University, Israel			
Hiba	Zayyad			Infectious Diseases Unit, Baruch Padeh Medical Center; the Azrieli Faculty of Medicine, Bar-Ilan University, Safed, Israel			
Oren	Zimhony			Kaplan Medical Center, Clalit Health Services, Israel; Faculty of Medicine, Hebrew University Jerusalem, Israel			
